# Components of Cell-Matrix Linkage as Potential New Markers for Prostate Cancer

**DOI:** 10.3390/cancers3010883

**Published:** 2011-02-25

**Authors:** Alexey Navdaev, Johannes A. Eble

**Affiliations:** Center for Molecular Medicine, Deptartment Vascular Matrix Biology, Excellence Cluster Cardio-Pulmonary System, Frankfurt University Hospital, Theodor-Stern-Kai 7, 60590 Frankfurt am Main, Germany; E-Mail: navdaev@med.uni-frankfurt.de

**Keywords:** prostate cancer, laminins, integrins, proteases, diagnosis

## Abstract

Prostate cancer is one of the most common tumor diseases worldwide. Often being non-aggressive, prostate tumors in these cases do not need immediate treatment. However, about 20% of diagnosed prostate cancers tend to metastasize and require treatment. Existing diagnostic methods may fail to accurately recognize the transition of a dormant, non-aggressive tumor into highly malignant prostate cancer. Therefore, new diagnostic tools are needed to improve diagnosis and therapy of prostate carcinoma. This review evaluates existing methods to diagnose prostate carcinoma, such as the biochemical marker prostate-specific antigen (PSA), but also discusses the possibility to use the altered expression of integrins and laminin-332 in prostate carcinomas as diagnostic tools and therapeutic targets of prostate cancer.

## 1. Prostate Carcinoma

Prostate carcinoma (PC) is one of the most frequent cancers diagnosed in men worldwide. For 2010, the American Cancer Society estimated 218,000 new cases of PC with 32,000 fatal cases among them. In Europe 338,000 new cases of PC were diagnosed in 2008 and more than 70,000 patients died. The frequency of PC is correlated to age. Nowadays, 0.1% of men in their 40s are diagnosed with PC, a frequency which rises to 8% at the age of 70 and older.

In most cases, PC shows very slow or even no growth over long time periods (ten years or more) and many patients diagnosed with such dormant PC do not need immediate treatment like surgery or irradiation. If a patient is older than 70 and the probability of the patient's death by reasons other than PC is relatively high, a strategy called active surveillance can be suggested [1]. In every individual case, the decision about the treatment strategy must be made by the attending physician, together with the patient, based on the clinicopathologic status of the disease. However, PC may develop a very aggressive and malignant behavior, which is characterized by fast growth of the primary tumor, dissemination of tumor cells from the primary tumor tissue and colonization of other organs. More than 80% of patients with metastasizing PC have metastases in bones and lymph nodes [2]. To date, the nature of this phenomenon is still poorly understood. It is discussed whether a specific extracellular microenvironment within these organs provides an appropriate substratum to which PC metastatic cells adhere to, and also whether PC cells develop the features mimicking osteoblasts [3,4]. Both hypotheses are supported by the finding that the number of PC-derived metastases is not increased after surgical treatment, which is normally accompanied by a significant release of PC cells into the blood circulation [5].

The factors which determine the transition of non-aggressive dormant nodes into very aggressive, highly malignant PC are not yet established. Moreover, the oncogenically transformed cells may be of different origin, which in turn affects their malignant behavior. Prostate epithelium consists of several cell types, including luminal, basal, intermediate, and neuroendocrine cells. PC can originate from luminal cells, and is then characterized by uncontrolled growth and the absence of intermingling basal cells [6]. Controversially, a recent report showed that human basal cells underwent oncogenic transformation and grew to tumors after transplantation into nude mice [7]. Therefore, it is possible that PC may originate from both luminal and basal cells.

Once awakened from their dormant form, malignant cells of PC are highly aggressive and need to be treated. There are several different treatments for PC. In most cases the preferred treatments are surgical dissection and radiotherapy. In some cases a hormone therapy can be used as well, but not all PC are sensitive to this method. Recently, several additional therapies have been developed and tested. In cryosurgery, tumors are destroyed by freezing. Biotherapy (immunotherapy) boosts the patient's immune system utilizing injected specific antigens. Furthermore, focused high-intensity ultrasound is used to treat PC. An active surveillance with routine clinical check-ups is recommended at an early stage of the disease when the tumor is small in size and does not show tendency to grow [8].

The total number of diagnosed PCs has dramatically increased over the last 20 years, whereas associated mortality did not change significantly. This discrepancy can be explained by the fact that new diagnostic methods allowed the detection of PCs at very early and curable stages, which had not been possible before. However, when a dormant node transforms into a malignant PC, the prognosis for the patient is still poor. Existing diagnostic methods often fail to detect this transition. Biochemical monitoring of prostate-specific antigen (PSA), together with digital rectal examination and invasive biopsy are the main tools to diagnose PC nowadays.

## 2. Existing Tools in PC Diagnostics

### 2.1. Prostate-specific Antigen (PSA)

In the late 1960s, a prostate specific antigen (PSA) was independently identified by several research groups and therefore named differently (kallikrein III, seminin, semenogelase, γ-seminoprotein and P-30 antigen). PSA is a 34 kDa serine protease which is secreted by cells of the prostate tissue. Later studies showed that the prostate is not the only source of PSA. However, as other organs produce PSA only in trace amounts, almost all PSA which circulates in the blood is the product of the prostate gland. The blood serum concentration of PSA is normally as low as just a few nanograms per milliliter. Yet, it can increase considerably or even by orders of magnitudes in advanced PC. Hence, PSA was proposed to be a suitable marker for early detection of PC. In the USA, a PSA test has been approved to detect prostate cancer in men aged 50 years and older. This test was also approved to monitor patients with a history of PC to survey cancer recurrence. However, there is the possibility of false positive results, because increased blood levels of PSA do not only occur in patients suffering from PC, but can also be caused by prostate infection, irritation, benign prostatic hyperplasia (BPH), and recent ejaculation. Conversely, this test can give false negative results as well, as a relatively high percentage of patients with PC do not show an increased blood level of PSA [9]. These contraries brought up the question whether the PSA assay is a reliable diagnostic method for PC and whether it can aid to decrease PC-associated mortality. To answer these questions several trials have been performed. Recently, results of two trials carried out in the USA and in Europe were published and show some controversy. In these trials, patients were annually examined for PSA blood level and biopsy was done for those with more than 4 ng/mL PSA. Patients with PSA levels between 3 and 4 ng/mL were additionally checked with digital rectal exam or transrectal ultrasound. According to the European data, the PSA-based screening reduced the PC-caused death rate by 20%, but it was associated with a high risk of overdiagnosis [10]. However, the data published by American researchers suggests that there is no significant decrease in PC-caused mortality in correlation with PSA screening [11]. These data, as well as data of several other randomized controlled trials, are reviewed in a very recent article by Schröder [12]. He reported that the thresholds of PSA blood levels, which are used as a cutoff in the assay, strongly influenced the screening results. He assumed that at the threshold of 4 ng/mL PSA about 75% of small PC escaped detection. When the threshold was decreased to 3 ng/mL, the percentage of non-diagnosed small PC dropped to 46%. Yet at the same time the number of false positive diagnoses dramatically increased.

Therefore, despite the fact that PSA screening can be helpful in PC diagnostics, it is not sufficient to recognize all cases of the disease and in most cases it must be assisted by additional, non-invasive tests, such as digital rectal examination, transrectal ultrasound examination, and histological analyses of biopsies.

### 2.2. Digital Rectal Examination and Transrectal Ultrasound

Digital rectal examination detects the swelling of the prostate gland. Together with the PSA test, it allows identification of the disease at relatively early stages. However, small tumors, which are typical for early stages of PC, are rarely detected by palpation. By transrectal ultrasound (TRUS), the entire prostate gland can be scrutinized. Thus, the size of the prostate and the presence of tumor nodes can be determined. Similar to digital rectal examination, TRUS bears the risk of overlooking small clusters of tumor cells. Mostly, ultrasound is used to visualize and control biopsy sample collection.

### 2.3. Biopsy

Biopsy is an invasive test in which a tissue sample is obtained with a needle and subsequently analyzed histologically. The histological evaluation employs the Gleason grading system, which was established in the 1960s with some modifications in the 1970s. According to the original procedure, cells of the prostate tumor tissue were assigned into five grades. Highly differentiated cells which resemble non-transformed cells are assigned to grade 1. Non-differentiated, highly modified cells that are likely aggressive with a high migratory activity are assigned to grade 5. By adding the grade numbers of the most common tumor patterns together, pathologists calculated the Gleason score, which ranged from 2 to 10. The great technical improvements for prostate surgery, biopsy, and histology achieved in the last decades provided new data which required modification of the existing Gleason grading system and interpretation of the results. Thus, the International Society of Urological Pathology (ISUP) reached a consensus about the changes which have to occur during analysis of biopsy samples by the Gleason grading system. According to the new recommendations, samples with a Gleason score of 2 should not be diagnosed as a low grade of tumor although there are some exceptions. When the Gleason score is 3 or 4, diagnosis can be made in rare cases and never without consultation. In case of a high Gleason score, the grade numbers of the most common tumor cells and the cells with the highest grade must be added to each other. More details on the consensus of the ISUP conference can be found in Epstein *et al.* [13]. Furthermore, the changes and suggestions concerning the improvement of the Gleason grading system are summarized in a recent article by Epstein [14].

Although the results of a biopsy may provide reliable data about a PC state, this method also has some shortcomings. Among them is the fact that the collected sample may not represent the entire organ. Usually, biopsy is performed under the control of TRUS, which helps to find the area where the sample is to be collected. However, due to an inhomogeneous distribution of malignant cells in a tumor tissue, the sample may not represent the actual situation in the tumor. A study by Robinson [15] reported that the examination of the surgically removed prostate showed a higher Gleason grade than was found during examination of a sample obtained by biopsy. In addition, results from histological examinations are subjective and depend on the personal experience of the pathologist. Therefore, the biopsy results could be improved by including independent quantitative markers into the PC diagnostic.

Thus, data obtained by several independent tests (*i.e.*, digital rectal examination, PSA value, transrectal ultrasound, biopsy) are taken in account to make a decision about the treatment strategy for each individual patient. However, the biochemical differences between non-aggressive and aggressive PCs are still not entirely clear. Several studies reported that PC cells of different malignancy states differ in their ability to express proteins involved in cell-matrix interaction, thus influencing cell migration. These are integrins, laminins and proteases, which are also involved in the process of extracellular matrix remodeling and degradation.

## 3. The Extracellular Matrix and Migration of Carcinoma Cells

**Figure 1.**
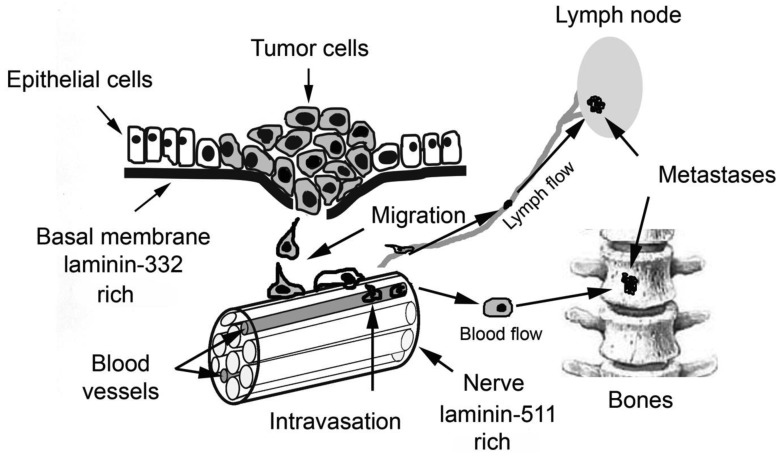
Metastatic cascade of prostate carcinoma (PC). PC cells grow into a primary tumor mostly within the peripheral zone of the prostate. At an aggressive stage, carcinoma cells start to disseminate from the primary tumor and penetrate through a basal membrane and stroma. After invading blood vessels or lymphatics, carcinoma cells can reach different organs where they can extravasate, settle, and form metastases, most often in bones and lymph nodes. Characteristically, PC cells also tend to migrate along nerve bundles which abundantly innervate the prostate.

Aggressive carcinoma cells migrate from the primary tumor, invade and infiltrate into neighboring tissues. After penetrating blood or lymphatic vessels they can colonize other organs which are located far from the primary tumor. Almost any step of this metastatic cascade requires contact to basal membranes, which are penetrated by the tumor cells (Figure 1).

Basal membranes (BMs) are thin layers of extracellular matrix proteins that constitute the borderline structure between connective tissue and any other tissue. Epithelial and endothelial cells are in close contact to the BM and are mechanically anchored via cell adhesion molecules to the BM. Muscle fibers, fat, and nerve cells are surrounded by a BM, which gives important environmental signals to these cells. Typical molecular components of the epithelial BM are collagen type IV and VII and laminins [16,17]. The dense meshwork of BM components is impermeable to most of the cells under normal conditions. Only leucocytes on their immunological patrol and pathologically malignant tumor cells are able to cross the BM. Key players involved in invasion of malignant cells through BMs are laminins (especially laminin-332), integrins, and proteases.

## 4. Laminins

Laminins are large glycoprotein molecules consisting of α, β and γ chains. There are five α, three β, and three γ chains identified to date. In different combinations they form 16 known laminin molecules, named according to their chain composition [18]. All laminins are characterized by a rod-like coiled-coil domain consisting of three chains, each folded individually into an α-helix. While the coiled-coil domain is visualized as a long arm in electron microscopic pictures, the N-terminal domains form the individual three short arms of the cruciform laminin molecule. Adjacent to the C-terminal end of the coiled-coil domain, the large globular (G) domain is composed solely of the laminin α chain and is further subdivided into five homologous LG subdomains, each about 200 amino acids in length [19]. The first three LG subdomains, LG1, LG2, and LG3, assemble into a clover leaf-like supradomain structure, which harbors the putative binding site for the laminin-binding integrins. However, the coiled-coil domain is necessary to form or stabilize the integrin-binding site within the G-domain [20].

Laminin-332 (previously known as laminin-5) consists of α-3, β-3 and γ-2 subunits. Laminin-332 interacts with collagen VII of BMs via a binding site located at the *N*-terminal end of the β-3 chain [21]. At the same time, it can interact with several cell surface receptors via its LG domains. Thereby, the cell and its cell adhesion molecules are connected to the BM with its force-bearing meshwork of laminins and collagen IV. Within the epidermal junctional zone, laminin-332 supramolecularly arranges into anchoring filaments which are found on the epithelial face of the BM (Lamina lucida) [22].

Laminin-332 can modulate migration of carcinoma cells. However, so far it has remained unclear whether laminin-332 accelerates or decelerates cell migration. Laminin-332 may provide strong cell binding and spreading, thus slowing down the motility of epithelial and carcinoma cells [23]. In contrast, laminin-332 may support migration and invasion of carcinoma cells [24,25]. This discrepancy might be explained by the fact that laminin-332 is processed by different proteases which are expressed in varying amounts by carcinoma cells. Several matrix metalloproteases (MMPs) [26] as well as plasmin [27,28] are responsible for these different proteolytic modifications of laminin-332, triggering distinct cellular functions. In addition to proteolytic modification, the effect of laminin-332 on cellular responses can be modulated by changes in expression of integrins binding laminin-332. It is known that α3β1, α6β1 and α6β4 integrins can all bind laminin-332 but induce different cellular effects. The α6β4 integrin induces strong cell adhesion upon binding to the laminin-332, whereas the α6β1 integrin promotes cell migration.

Laminin-511 (previously known as laminin-10) consists of α5, β1, and γ1 chains. The α5 chain is longer than α3 due to several additional *N*-terminal domains. The G domain of laminin-511 can interact with α3β1, α6β1, and α6β4 integrins. It is not yet clear whether laminin-511 and laminin-332 use the same or different binding sites of these receptors. The β1 and γ1 chains cannot be digested by MT1-MMP in contrast to β3 and γ2. However, MT1-MMP digests the α5-chain, which promotes PC cell migration [29]. Enhanced migration of carcinoma cells in this case can be explained by decreasing cell adhesion which slows migration. To date, there is no published data showing an active modulating role of laminin-511 in carcinoma cell migration as is the case for laminin-332.

## 5. Integrins

Integrins are a large family of cell surface molecules involved in adhesion, cell-cell interaction and regulation of cell growth and migration. They are heterodimeric molecules consisting of α and β chains. Up to date, 18 α subunits and 8 β subunits have been identified which combine into 24 known integrins. The integrins α3β1, α7β1, α6β1 and α6β4 interact with laminins. The integrins α6β4 and α3β1 show specificity to laminin-332 and laminin-511, whereas α7β1 binds to laminin α1, α2 or α5 chain-containing laminins [30]. The integrin α6β1 shows a broader specificity and can bind to several laminin isoforms. Upon binding to laminin-332 the integrin α6p4 can organize hemidesmosomes, which are essential for stable cell adhesion to BM [31]. The α6p1 integrin is involved in cell migration [32]. The role of the integrin α3β1 in cell migration is ambivalent. Whereas some publications report that this integrin stabilizes cell adhesion and inhibits cell migration upon binding to laminin-332 [33], other studies ascribe a promigratory role to α3β1 integrin in epithelial cells [34]. This discrepancy cannot be explained yet and may depend on the cell type and/or additional signals within the cell. All integrins are transmembrane receptors and, along with other signal-transducing molecules, relay signals from the extracellular matrix (ECM) into the cell. Cross-talk of these signals with cues from growth factor receptors and further signaling results in cell-type specific reactions [35]. In a recent study, Edick *et al.* [36] showed that α3β1 integrin can prevent cell death of normal basal prostate cells by activating EGFR/Erk but not PI3-K signaling and/or autophagy activation. However, in PC3 cells α3β1 integrin provides cell survival through activation of PI3-K. In both case, signaling events are initiated upon binding of α3β1 integrin to laminin-332. It is not yet clear whether the same signaling pathways are involved in PC cell migration on laminin-511 which is also a ligand for α3β1 integrin.

## 6. Proteases

To be able to move, migrating cells secrete a number of proteases, among them matrix metalloproteases (MMPs) which cleave ECM proteins, thereby breaking links between cell surface receptors and extracellular matrix proteins [37]. MT1-MMP, a membrane bound form of MMPs, is a key enzyme in the process of cancer cell invasion. MT1-MMP is responsible for cleaving basement membrane proteins and type I collagen in the stromal connective tissue, both necessary steps in tumor cell invasion. Upregulation of MT1-MMP was found in almost all invading carcinoma cells [38]. In addition to MMPs, several other proteases are secreted by malignant cells, e.g. matriptase and hepsin. Both are members of the type II transmembrane serine protease family and are abundantly produced by PC cells [39,40]. The metalloproteinase ADAM15 was also found to be upregulated in PC and its role as a regulatory component of tumor progression and its possible use as a biomarker is discussed [41]. Despite the fact that many membrane-bound and soluble proteases are overexpressed by PC, the MT1-MMP probably is the most important for ECM degradation and modulation of migratory behavior of carcinoma cells.

## 7. The Interplay of Laminin, Integrins and Proteases in PC Metastasis

Enhanced expression of the laminin-332 or its individual subunits by many different carcinomas was shown *in vitro* and *in vivo* [42,43]. Laminin-332 stimulates carcinoma cells to form lamellipodia and enhances cell migration. During cell migration, cells must lose certain cell-matrix contacts and detach from the surrounding matrix [44]. Proteases released by migrating malignant cells can degrade the pericellular matrix proteins, thereby destroying firm cell anchorage points. However, complete detachment of cells would hamper cell migration, as the ECM meshwork also serves as a substrate to which cells attach and from which cells receive environmental and survival cues. Tumor cells may compensate for these lost contacts by enhanced secretion of extracellular proteins. In fact, binding of laminin-332 to α3β1 integrin increases cell viability by triggering PI3-K, 14-3-3, and FAK signaling cascades, which eventually lead to suppression of caspase activation [45]. Also, it was found that the fragment of the γ2 chain of laminin-332 can bind to the EGF receptor and enhance cell motility [46,47].

In contrast to many other carcinomas, PC typically produces less laminin-332 and its cognate receptor, α6β4 integrin [48,49]. In most cases of PC, laminin-332 expression is not completely absent because neighboring non-transformed basal prostate cells still produce laminin-332, which allows prostate carcinoma cells to survive and migrate [50].

In a recent study, a subpopulation of cells named TEM4-18 was isolated from PC-3, a prostate carcinoma cell line. This newly identified subpopulation of cells is characterized by overexpression of ZEB-1, a known regulator of epithelial-to-mesenchymal transition, and was able to intra- and extravasate through endothelial barriers more efficiently than parental PC-3 cells. Also, TEM4-18 exhibited enhanced metastatic colonization in a murine *in vivo* model. Surprisingly, TEM4-18 cells were less invasive than parental PC-3 cells in *in vitro* assays involving matrix barriers [51]. In the most recent work, it was shown that highly aggressive TEM4-18 cells strongly reduce laminin-332 expression in comparison to less aggressive PC-3 cells which still produce a moderate level of this protein. The invasiveness of TEM4-18 cells *in vitro* was restored when exogenous laminin-332 was added or when cells were co-cultured with laminin-332 secreting cells [52]. Thus, these highly aggressive PC cells still needed interaction with laminin-332 to be able to migrate despite the fact that they did not produce it by themselves.

This discrepancy is nevertheless well correlated with data concerning the role of laminin-332 in carcinoma cell migration. On the one hand, it is shown that laminin-332 is over-expressed by many carcinomas and accelerates migration of malignant cells. On the other hand, numerous accounts confirm reduction of laminin-332 expression by PC. It can be assumed that highly invasive PC cells, which show reduced expression of laminin-332 during migration can use the protein secreted by neighboring non-invasive cells. Moreover, during migration aggressive PC cells can use laminin-511 whose expression by these cells is not decreased. Also, migration of PC cells along the nerve bundles, which have laminin-511 enriched basal membranes, is well known.

The mechanism regulating the expression of laminin-332 in PC cells is not fully understood. ZEB1, a regulator of epithelial-to-mesenchymal transition, regulates laminin-332 and β4 integrin expression [52]. However, other, still unknown factors may also affect laminin-332 synthesis. Moreover, the strong lack of laminin-332 may be aggravated by its degradation by proteases. Increased mobility of PC cells is likely related to the proteolytic processing of laminin-332 by secreted proteases [40,53].

Laminin-511 is abundant in basement membranes ensheathing nerves, which numerously innervate the prostate gland [32,54]. Typically, PC cells disseminate from the peripheral zone of the gland along nerve bundles which reach the prostate capsule [55,56]. During this perineural invasion (PNI) several mediators and chemokines are released by both the cancer and the nerve cells which stimulate tumor cell survival and facilitate their migration [57]. Laminin-511 of the perinerium is recognized by α6β1 integrin, and thus plays an important role in PNI [32]. The expression of laminin-511 is not altered in prostate cancer but its ability to interact with integrins can be strongly decreased due to α5 chain processing by MT1-MMP which is over-expressed by carcinoma cells [29].

In contrast to laminin-332 and its cognate receptor α6β4 integrin, the expression of the laminin receptor α6β1 is unaltered or even upregulated [58]. The α6β1 integrin binds to laminin-511 and mediates cell migration [30]. In PC, α6β1 integrin expression is strongly reduced while the α6β1 integrin level does not changed. There while, the question about α3β1 integrin expression in PC remains open. There are only few articles regarding α3β1 expression with very controversial results. In a recent article by Schmelz *et al.* [59], the authors reported that low Gleason sum score is associated with increased expression of the integrin. The samples with a high Gleason sum score showed very low or even an absence of the α3β1 expression. In another article, the authors conclude that an increase in α3β1 expression is typical for highly aggressive PC cells and associated with a higher risk for cancer recurrence [60]. Thus, α3β1 integrin expression by PC cells requires further examination.

## 8. Conclusions

During transformation from a non-invasive to a highly malignant phenotype, PC changes the expression levels of several proteins involved in cell-matrix interactions. The laminin-332 level is down-regulated in contrast to the unaltered expression of laminin-511. The integrin α6β4 expression is down-regulated while integrin α6β1 is not changed or is even upregulated. There is no uniform opinion about the expression of integrin α3β1 by PC cells and this issue needs further investigation. PC cells show elevated level of MT1-MMP and secreted matriptase. Quantification of these changes could provide a PC-typical tumor signature and may improve the diagnostics of PC, which so far has been based on histological examination of biopsies and the Gleason score calculation, along with the blood marker PSA. Moreover, understanding the underlying mechanisms of how these molecules target and mediate migration, invasion, and progression of prostate cancer cells may open new pharmaceutical avenues to treat PC.

## Figures and Tables

**Figure 2.**
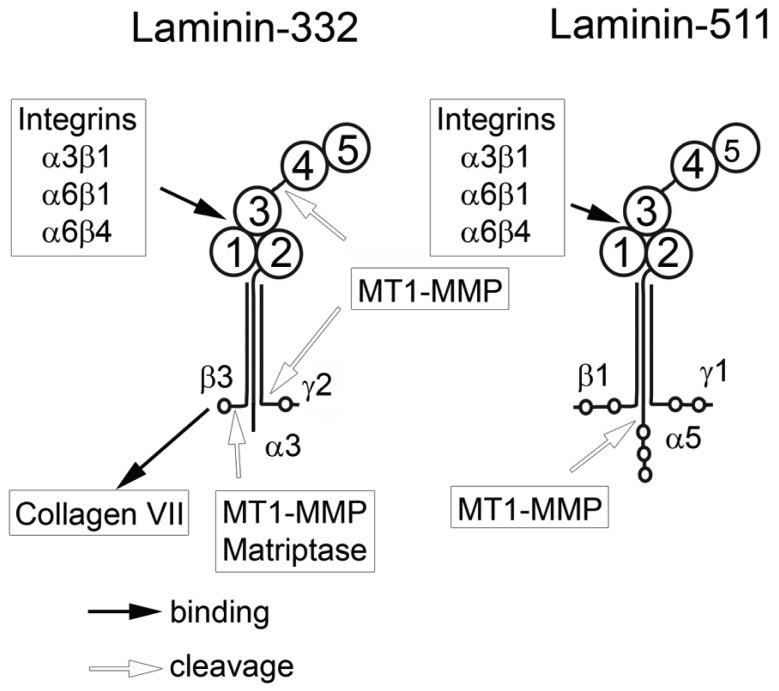
Structures of the non-processed laminin-332 and laminin-511. A characteristic hallmark of laminins is the α-helical coiled-coil domain consisting of three laminin chains, α3β3γ2 or α5β1γ1 for laminin-332 and laminin-511, respectively. Each chain can be cleaved by several different proteases with drastic effects on its biological functions. Exclusively formed by the *C*-terminal portion of the α3 chain, the globular (G) domain can be subdivided into five LG-domains, each about 200 amino acids in length. The LG4-5 tandem domain is connected to the LG1-3 domain cluster via connecting sequence. The connecting sequence contains target sites for several proteases. The G-domain contains the putative binding site for the integrins α3β1, α6β1, and α6β4. Secreted laminin-332 undergoes fast processing by proteases which cleave the LG4-5 domain of the α3 chain and the *N*-terminal domain of the γ2 chain.

## References

[1] Albertsen P.C. (2010). Treatment of localized prostate cancer: When is active surveillance appropriate?. Nat. Rev. Clin. Oncol..

[2] Harada M., Iida M., Yamaguchi M., Shida K. (1992). Analysis of bone metastasis of prostatic adenocarcinoma in 137 autopsy cases. Adv. Exp. Med. Biols..

[3] Rucci N., Teti A. (2010). Osteomimicry: How tumor cells try to deceive the bone. Front Biosci. (Schol. Ed.).

[4] Datta K., Muders M., Zhang H., Tindall D.J. (2010). Mechanism of lymph node metastasis in prostate cancer. Future Oncol..

[5] Morgan T.M., Lange P.H., Porter M.P., Lin D.W., Ellis W.J., Gallaher I.S., Vessella R.L. (2009). Disseminated tumor cells in prostate cancer patients after radical prostatectomy and without evidence of disease predicts biochemical recurrence. Clin. Cancer Res..

[6] Wang X., Kruithof-de Julio M., Economides K.D., Walker D., Yu H., Halili M.V., Hu Y.P., Price S.M., Abate-Shen C., Shen M.M. (2009). A luminal epithelial stem cell that is a cell of origin for prostate cancer. Nature.

[7] Goldstein A.S., Huang J., Guo C., Garraway I.P., Witte O.N. (2010). Identification of a cell of origin for human prostate cancer. Science.

[8] Dall’Era M.A., Konety B.R., Cowan J.E., Shinohara K., Stauf F., Cooperberg M.R., Meng M.V., Kane C.J., Perez N., Master V.A., Carroll P.R. (2008). Active surveillance for the management of prostate cancer in a contemporary cohort. Cancer.

[9] Thompson I.M., Pauler D.K., Goodman P.J., Tangen C.M., Lucia M.S., Parnes H.L., Minasian L.M., Ford L.G., Lippman S.M., Crawford E.D., Crowley J.J., Coltman C.A. (2004). Prevalence of prostate cancer among men with a prostate-specific antigen level < or =4.0 ng per milliliter. N Engl. J. Med..

[10] Schroder F.H., Hugosson J., Roobol M.J., Tammela T.L., Ciatto S., Nelen V., Kwiatkowski M., Lujan M., Lilja H., Zappa M., Denis L.J., Recker F., Berenguer A., Maattanen L., Bangma C.H., Aus G., Villers A., Rebillard X., van der Kwast T., Blijenberg B.G., Moss S.M., de Koning H.J., Auvinen A. (2009). Screening and prostate-cancer mortality in a randomized european study. N. Engl. J. Med..

[11] Andriole G.L., Crawford E.D., Grubb R.L., Buys S.S., Chia D., Church T.R., Fouad M.N., Gelmann E.P., Kvale P.A., Reding D.J., Weissfeld J.L., Yokochi L.A., O’Brien B., Clapp J.D., Rathmell J.M., Riley T.L., Hayes R.B., Kramer B.S., Izmirlian G., Miller A.B., Pinsky P.F., Prorok P.C., Gohagan J.K., Berg C.D. (2009). Mortality results from a randomized prostate-cancer screening trial. N. Engl. J. Med..

[12] Schroder F.H. (2010). Prostate cancer around the world. An overview. Urol. Oncol..

[13] Epstein J.I., Allsbrook W.C., Amin M.B., Egevad L.L. (2005). The 2005 international society of urological pathology (ISUP) consensus conference on gleason grading of prostatic carcinoma. Am. J. Surg. Pathol..

[14] Epstein J.I. (2010). An update of the gleason grading system. J. Urol..

[15] Robinson B.D., Epstein J.I. (2010). Intraductal carcinoma of the prostate without invasive carcinoma on needle biopsy: Emphasis on radical prostatectomy findings. J. Urol..

[16] Nishiyama T., Amano S., Tsunenaga M., Kadoya K., Takeda A., Adachi E., Burgeson R.E. (2000). The importance of laminin 5 in the dermal-epidermal basement membrane. J. Dermatol. Sci..

[17] Yurchenco P.D., O’Rear J.J. (1994). Basal lamina assembly. Curr. Opin. Cell Biol..

[18] Aumailley M., Bruckner-Tuderman L., Carter W.G., Deutzmann R., Edgar D., Ekblom P., Engel J., Engvall E., Hohenester E., Jones J.C., Kleinman H.K., Marinkovich M.P., Martin G.R., Mayer U., Meneguzzi G., Miner J.H., Miyazaki K., Patarroyo M., Paulsson M., Quaranta V., Sanes J.R., Sasaki T., Sekiguchi K., Sorokin L.M., Talts J.F., Tryggvason K., Uitto J., Virtanen I., von der Mark K., Wewer U.M., Yamada Y., Yurchenco P.D. (2005). A simplified laminin nomenclature. Matrix. Biol..

[19] Timpl R., Tisi D., Talts J.F., Andac Z., Sasaki T., Hohenester E. (2000). Structure and function of laminin lg modules. Matrix. Biol..

[20] Kunneken K., Pohlentz G., Schmidt-Hederich A., Odenthal U., Smyth N., Peter-Katalinic J., Bruckner P., Eble J.A. (2004). Recombinant human laminin-5 domains. Effects of heterotrimerization, proteolytic processing, and *N*-glycosylation on alpha3beta1 integrin binding. J. Biol. Chem..

[21] Nakashima Y., Kariya Y., Yasuda C., Miyazaki K. (2005). Regulation of cell adhesion and type vii collagen binding by the beta3 chain short arm of laminin-5: Effect of its proteolytic cleavage. J. Biochem..

[22] Rousselle P., Keene D.R., Ruggiero F., Champliaud M.F., Rest M., Burgeson R.E. (1997). Laminin 5 binds the NC-1 domain of type vii collagen. J. Cell Biol..

[23] Yuen H.W., Ziober A.F., Gopal P., Nasrallah I., Falls E.M., Meneguzzi G., Ang H.Q., Ziober B.L. (2005). Suppression of laminin-5 expression leads to increased motility, tumorigenicity, and invasion. Exp. Cell Res..

[24] Tani T., Lumme A., Linnala A., Kivilaakso E., Kiviluoto T., Burgeson R.E., Kangas L., Leivo I., Virtanen I. (1997). Pancreatic carcinomas deposit laminin-5, preferably adhere to laminin-5, and migrate on the newly deposited basement membrane. Am J. Pathol..

[25] Lohi J. (2001). Laminin-5 in the progression of carcinomas. Int. J. Cancer.

[26] Pirila E., Sharabi A., Salo T., Quaranta V., Tu H., Heljasvaara R., Koshikawa N., Sorsa T., Maisi P. (2003). Matrix metalloproteinases process the laminin-5 gamma 2-chain and regulate epithelial cell migration. Biochem. Biophys. Res. Commun..

[27] Goldfinger L.E., Stack M.S., Jones J.C. (1998). Processing of laminin-5 and its functional consequences: Role of plasmin and tissue-type plasminogen activator. J. Cell Biol..

[28] Ogura Y., Matsunaga Y., Nishiyama T., Amano S. (2008). Plasmin induces degradation and dysfunction of laminin 332 (laminin 5) and impaired assembly of basement membrane at the dermal-epidermal junction. Br. J. Dermatol..

[29] Bair E.L., Chen M.L., McDaniel K., Sekiguchi K., Cress A.E., Nagle R.B., Bowden G.T. (2005). Membrane type 1 matrix metalloprotease cleaves laminin-10 and promotes prostate cancer cell migration. Neoplasia.

[30] Nishiuchi R., Takagi J., Hayashi M., Ido H., Yagi Y., Sanzen N., Tsuji T., Yamada M., Sekiguchi K. (2006). Ligand-binding specificities of laminin-binding integrins: A comprehensive survey of laminin-integrin interactions using recombinant alpha3beta1, alpha6beta1, alpha7beta1 and alpha6beta4 integrins. Matrix Biol.

[31] Litjens S.H., de Pereda J.M., Sonnenberg A. (2006). Current insights into the formation and breakdown of hemidesmosomes. Trends Cell Biol..

[32] Sroka I.C., Anderson T.A., McDaniel K.M., Nagle R.B., Gretzer M.B., Cress A.E. (2010). The laminin binding integrin alpha6beta1 in prostate cancer perineural invasion. J. Cell Physiol..

[33] Margadant C., Raymond K., Kreft M., Sachs N., Janssen H., Sonnenberg A. (2009). Integrin alpha3beta1 inhibits directional migration and wound re-epithelialization in the skin. J. Cell Sci..

[34] Choma D.P., Pumiglia K., DiPersio C.M. (2004). Integrin alpha3beta1 directs the stabilization of a polarized lamellipodium in epithelial cells through activation of rac1. J. Cell Sci..

[35] Hynes R.O. (2002). Integrins: Bidirectional, allosteric signaling machines. Cell.

[36] Edick M.J., Tesfay L., Lamb L.E., Knudsen B.S., Miranti C.K. (2007). Inhibition of integrin-mediated crosstalk with epidermal growth factor receptor/Erk or src signaling pathways in autophagic prostate epithelial cells induces caspase-independent death. Mol. Biol. Cell.

[37] Chen P., Parks W.C. (2009). Role of matrix metalloproteinases in epithelial migration. J. Cell Biochem..

[38] Poincloux R., Lizarraga F., Chavrier P. (2009). Matrix invasion by tumour cells: A focus on mt1-mmp trafficking to invadopodia. J. Cell Sci..

[39] Tripathi M., Nandana S., Yamashita H., Ganesan R., Kirchhofer D., Quaranta V. (2008). Laminin-332 is a substrate for hepsin, a protease associated with prostate cancer progression. J. Biol. Chem..

[40] Tripathi M., Potdar A.A., Yamashita H., Weidow B., Cummings P.T., Kirchhofer D., Quaranta V. (2010). Laminin-332 cleavage by matriptase alters motility parameters of prostate cancer cells. Prostate.

[41] Lucas N., Day M.L. (2009). The role of the disintegrin metalloproteinase adam15 in prostate cancer progression. J. Cell Biochem..

[42] Miyazaki K. (2006). Laminin-5 (laminin-332): Unique biological activity and role in tumor growth and invasion. Cancer Sci..

[43] Guess C.M., Quaranta V. (2009). Defining the role of laminin-332 in carcinoma. Matrix. Biol..

[44] Ridley A.J., Schwartz M.A., Burridge K., Firtel R.A., Ginsberg M.H., Borisy G., Parsons J.T., Horwitz A.R. (2003). Cell migration: Integrating signals from front to back. Science.

[45] Oh J.E., Jang da H., Kim H., Kang H.K., Chung C.P., Park W.H., Min B.M. (2009). Alpha3beta1 integrin promotes cell survival via multiple interactions between 14-3-3 isoforms and proapoptotic proteins. Exp. Cell Res..

[46] Schenk S., Hintermann E., Bilban M., Koshikawa N., Hojilla C., Khokha R., Quaranta V. (2003). Binding to EGF receptor of a laminin-5 EGF-like fragment liberated during mmp-dependent mammary gland involution. J. Cell Biol..

[47] Koshikawa N., Schenk S., Moeckel G., Sharabi A., Miyazaki K., Gardner H., Zent R., Quaranta V. (2004). Proteolytic processing of laminin-5 by mt1-mmp in tissues and its effects on epithelial cell morphology. FASEB J..

[48] Hao J., Jackson L., Calaluce R., McDaniel K., Dalkin B.L., Nagle R.B. (2001). Investigation into the mechanism of the loss of laminin 5 (alpha3beta3gamma2) expression in prostate cancer. Am. J. Pathol..

[49] Davis T.L., Cress A.E., Dalkin B.L., Nagle R.B. (2001). Unique expression pattern of the alpha6beta4 integrin and laminin-5 in human prostate carcinoma. Prostate.

[50] Yu H.M., Frank D.E., Zhang J., You X., Carter W.G., Knudsen B.S. (2004). Basal prostate epithelial cells stimulate the migration of prostate cancer cells. Mol. Carcinog..

[51] Drake J.M., Strohbehn G., Bair T.B., Moreland J.G., Henry M.D. (2009). Zeb1 enhances transendothelial migration and represses the epithelial phenotype of prostate cancer cells. Mol. Biol. Cell.

[52] Drake J.M., Barnes J.M., Madsen J.M., Domann F.E., Stipp C.S., Henry M.D. (2010). Zeb1 coordinately regulates laminin-332 and {beta}4 integrin expression altering the invasive phenotype of prostate cancer cells. J. Biol. Chem..

[53] Udayakumar T.S., Chen M.L., Bair E.L., Von Bredow D.C., Cress A.E., Nagle R.B., Bowden G.T. (2003). Membrane type-1-matrix metalloproteinase expressed by prostate carcinoma cells cleaves human laminin-5 beta3 chain and induces cell migration. Cancer Res..

[54] Nagle R.B., Hao J., Knox J.D., Dalkin B.L., Clark V., Cress A.E. (1995). Expression of hemidesmosomal and extracellular matrix proteins by normal and malignant human prostate tissue. Am. J. Pathol..

[55] Villers A., McNeal J.E., Redwine E.A., Freiha F.S., Stamey T.A. (1989). The role of perineural space invasion in the local spread of prostatic adenocarcinoma. J. Urol..

[56] Liebig C., Ayala G., Wilks J., Verstovsek G., Liu H., Agarwal N., Berger D.H., Albo D. (2009). Perineural invasion is an independent predictor of outcome in colorectal cancer. J. Clin. Oncol..

[57] Marchesi F., Piemonti L., Mantovani A., Allavena P. (2010). Molecular mechanisms of perineural invasion, a forgotten pathway of dissemination and metastasis. Cytokine Growth Factor Rev..

[58] Goel H.L., Li J., Kogan S., Languino L.R. (2008). Integrins in prostate cancer progression. Endocr. Relat Cancer.

[59] Schmelz M., Cress A.E., Scott K.M., Burger F., Cui H., Sallam K., McDaniel K.M., Dalkin B.L., Nagle R.B. (2002). Different phenotypes in human prostate cancer: Alpha6 or alpha3 integrin in cell-extracellular adhesion sites. Neoplasia.

[60] Pontes-Junior J., Reis S.T., de Oliveira L.C., Sant’anna A.C., Dall’oglio M.F., Antunes A.A., Ribeiro-Filho L.A., Carvalho P.A., Cury J., Srougi M., Leite K.R. (2010). Association between integrin expression and prognosis in localized prostate cancer. Prostate.

